# Wound Myiasis in a Flamingo (*Phoenicopterus ruber*) Caused by *Calliphora* spp. Larvae from Northwest of Iran: A Case Report

**Published:** 2018

**Authors:** Roghayeh NOROUZI, Saeed KAHNAMOOIE, Ali OZBANDI

**Affiliations:** 1. Dept. of Pathobiology, Faculty of Veterinary Medicine, University of Tabriz, Tabriz, Iran; 2. Dept. of Tabriz City Environment Protection, Tabriz, Iran

**Keywords:** Wound myiasis, Flamingo (*Phoenicopterus ruber*), *Calliphora* spp., Northwest Iran

## Abstract

Myiasis is the infestation of animals or man tissues by parasitic dipterous fly larvae. Wound myiasis is the result of fly egg deposition on decaying flesh or pus discharging wounds. This case report describes a type of wound myiasis caused by *Calliphora* spp. in a Flamingo (*Phoenicopterus ruber)* from East Azerbaijan Province, Iran. A 3-yr-old female Flamingo was suffering in its left wing leading to an extensive discharging wound, which was heavily infested by maggots (fly larvae). The examination of external morphological characters of the second and third-instar larvae, posterior spiracles and internal cephalopharyngeal skeleton, led to the identification of the *Calliphora* spp. fly genus. Treatment consisted of removal of the larvae and surgical debridement, then spray of antibiotic and toxic drug. Following removal of larvae and treatment, the symptoms completely resolved within the last hour and remained asymptomatic several weeks later. This is the first report of wound myiasis in a Flamingo (*Phoenicopterus ruber)* by the facultative myiasis agent *Calliphora* spp. in Iran and the world.

## Introduction

Myiasis is the infestation of tissues of animals or man by the larvae of flies. Although, the most common site of myiasis is the skin wound, less common sites are the alimentary canal, eyes, ears, sinuses, throat, nose, and genitourinary tract (1). Wound myiasis is the result of egg deposition on open wounds with necrotic (prematurely dying) tissue or pus-discharging. Occasionally the maggots invade on superficial layers of exposed tissue and can develop subcutaneous nodules (2). Wound myiasis has a worldwide distribution because numerous genera and species have been implicated in cutaneous disease. Wild birds are susceptible to facultative and obligatory wound myiasis (2). The numerous of avian species, together with a wide range of presenting subjects, makes the majority of avian wound management a very complex problem (3). Many birds of the animal kingdom will suffer wing injuries during the course of their lives.

The larvae of Calliphoridae family, also known as the blowflies, are characterized to develop in animal flesh. “Species associated with an ectoparasitic lifestyle can be divided generally into three groups based on their larval feeding habits: saprophagy, facultative ectoparasitism, and obligate parasitism” (4). Flamingo or flamingoes belong to the Phoenicopteridae family (the only family in the order Phoenicopteriformes). The wound myiasis rarely affects birds, especially Flamingoes.

This case report describes a type of wound myiasis caused by *Calliphora* spp. in a Flamingo (*Phoenicopterus ruber)* with a wing injury history, from East Azerbaijan Province, Iran. Following removal of the larvae and surgical debridement healing progressed rapidly and within six days the wound was virtually healed.

## Case report

**Fig. 1:**
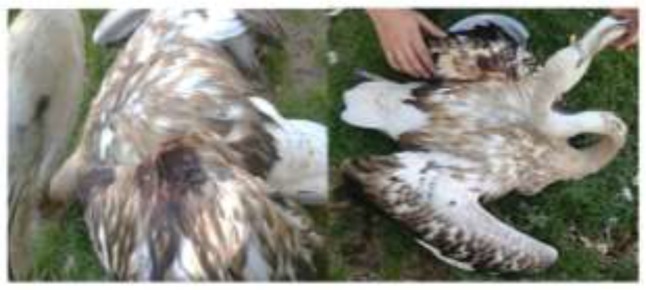
The injured Flamingo (*Phoenicopterus ruber)* in the East Azerbaijan Province, Iran, Sep 2, 2016

A 3-yr-old female Flamingo (*P. ruber)* with a specific wound in left wing was referred by the environmental department of East Azerbaijan Province to the private clinic of Tabriz City Environment Protection Department, in Northwest Iran in September 2016 (Fig. 1).

At the initial examination, clinical signs were extended with a wound upper the left wing.

The wound was infested with the numerous white maggots. The maggots were carefully removed from her wing using sterile forceps and placed in 10% neutral-buffered formalin. The numerous cylindrical vermiform maggot measuring 4-6 mm in length and 3 mm in diameter was observed under the dissecting microscope. The specimen was gently washed in phosphate-buffered saline, pH 7.4, and cleared in graded solutions of glycerol (up to 80%).

**Fig. 2:**
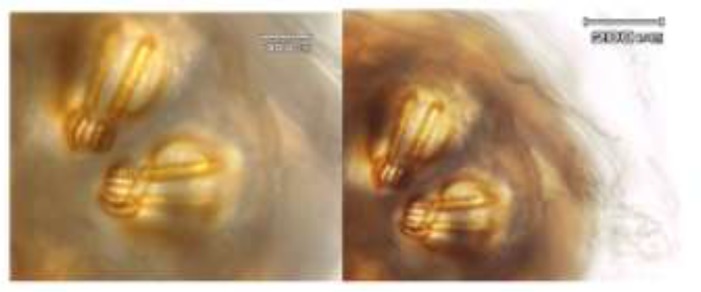
Posterior spiracle of *Calliphora* spp. larvae, 20X

**Fig. 3:**
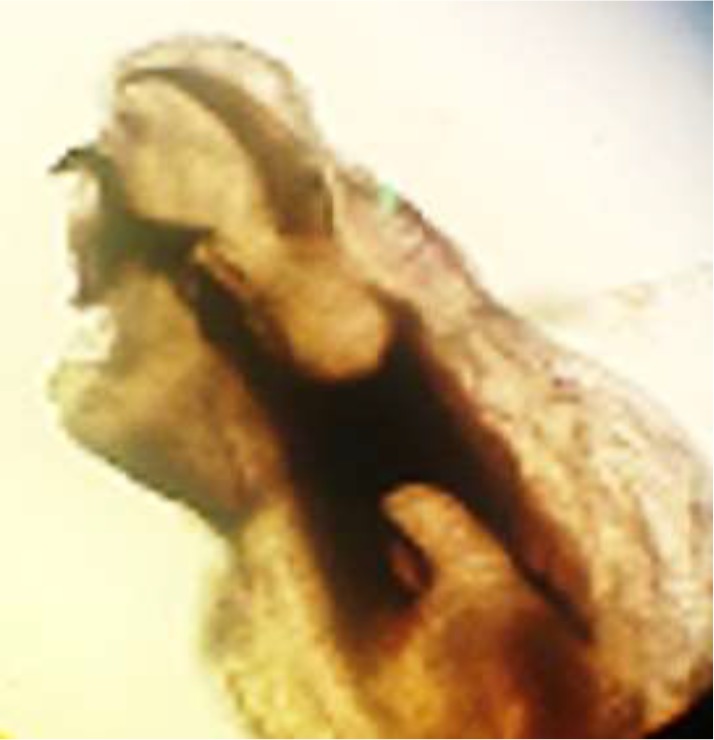
Anterior end of larva and terminal oral hooks of *Calliphora* spp. larvae, 40X

According to key diagnostic features for maggots in birds (5), the larvae were identified as second and third instars of *Calliphora* spp. has the posterior spiracles (Fig. 2). The cephaloskeleton was also large and darkly colored (Fig. 3).

## Discussion

Myiasis is a term used for the infestation of living human or animal tissues by the larvae of dipterous flies that, at least for a certain period, feed on the host’s necrotic or living tissue, liquid and discharge body, or ingested food and water (6). Wound myiasis has a worldwide distribution because numerous genera and species have been implicated in dermal disease. Flies causing myiasis to belong to the family of Diptera and its seven different species (Sarcophagidae, Gasterophylidae, Calliphoridae, Oestridae, Hypodermatidae, Muscidae and Glossinidae) have been known to invade the skin (7).

Myiasis in birds has been reported in geese due to the *L. sericata* and *L. cuprina* (8), in duck due to Sarcophagidae (9), in finch by Muscidae (10), in hawk by Protocalliphora (11), in nestling birds by *Protocalliphora braueri* larvae (12), and in owls by Muscidae and *Protocalliphora avium* (11, 13), in *Dendroica castanea* due to *Philornis* spp. (14), in geese due to *Wohlfahrtia magnifica* and *Lucilia sericata* (15) and in turkey by cutaneous myiasis by *L. sericata* and *L. cuprina* (16).

Wild birds wound myiasis has been reported in Eastern imperial eagle due to the *Calliphora vicina* from Khuzestan Province (Southwestern Iran) (17) and in owl due to the *Lucillia* spp. from Chaharmahal and Bakhtiari Province (west-central Iran) (18).

However, this report is the first report of wound myiasis in a Flamingo (*Phoenicopterus ruber)* by the facultative myiasis agent *Calliphora* spp. in Iran and the world.

## Conclusion

The myiasis should be considered as an accidental disease among wild bird because parasitic dipterous fly larvae infestations, especially Calliphoridae family, by endangering the health of wild birds cause be their threatened life. Awareness of the wound myiasis in protected area, especially during spring and summer, leads to the more prompt diagnosis, and institution of specific therapy for the disease.
